# 939 Impact of Sleep Disorders on Neurological Outcomes in Burn Survivors

**DOI:** 10.1093/jbcr/iraf019.470

**Published:** 2025-04-01

**Authors:** Rashid Syed, Jasmine Chaij, Juquan Song, Steven Wolf, George Golovko, Amina El Ayadi

**Affiliations:** University of Texas Medical Branch; University of Texas Medical Branch; University of Texas Medical Branch; University of Texas Medical Branch; University of Texas Medical Branch; University of Texas Medical Branch

## Abstract

**Introduction:**

Burn injuries are traumatic events that severely impact patients socially, psychologically, and physically. Some burn survivors tend to have more arduous recoveries with lasting impacts long after the primary injury. Altered neurologic outcomes after burn have been reported in some studies; however, it is not clear what factors predispose certain burn patients to poor neurologic outcomes. We investigated the impact of primary sleep disorder after a burn injury on neurologic outcomes.

**Methods:**

Using the TriNetX database, a large, federated research network of de-identified patient data, we identified patients with burns and compared those who developed a first-time sleep disorder after a burn to those who did not. Cohorts were propensity-matched by age, gender, race, ethnicity, and percent Total Body Surface Area (TBSA) burn. Excluding patients with pre-existing neurologic disorders, we evaluated new incidences of neurologic disorders including dementia, delirium, demyelinating disorders, seizures and epilepsy, migraines, mono- and polyneuropathies, and neurodegenerative disorders.

**Results:**

Of the 657,108 burn patients identified in TriNetX, 62,667 patients developed a sleep disorder post-burn. The age of patients developing a sleep disorder post-burn were significantly older than those who did not (43.8 ± 21.4 vs. 30.2 ± 22.3, p < 0.001). Neurodegenerative disorders were 2.29, 95% CI [2.21, 2.49] times more likely to occur if patients with burn injury developed a sleep disorder. Patients who developed a first-time sleep disorder after a burn showed a more than twofold increase in new neurologic diagnoses such as delirium, demyelinating disorder, migraines, neuropathies, and vascular dementia. Additionally, patients who developed a first-time sleep disorder were two times more likely to develop seizures and epilepsy 95% CI [1.83, 2.17] as well as unspecified dementia all with p=0.05. Most primary neurologic disorders occur later than 12 months after the onset of primary sleep disorder after a burn.

**Conclusions:**

The development of a sleep disorder after primary burn injury significantly increases the risk of subsequent poor neurologic outcomes. The post-burn period is integral for adequate neurologic recovery, and we demonstrate the importance of sleep in that process. This study aims to emphasize the importance of adequate sleep in improving neurologic outcomes after a burn.

**Applicability of Research to Practice:**

The majority of these disorders occurred more than 12 months after the onset of a primary sleep disorder after burn. This study underscores the long-term impact of burns and the integral role that sleep plays in neurologic recovery during the post-burn phase. We emphasize the importance of identifying and treating sleep disorders in burn survivors to mitigate poor neurologic outcomes.

**Funding for the Study:**

Database funding by National Center for Advancing Translational Sciences

